# Validation of a Behavior Test for Predicting Puppies’ Suitability as Detection Dogs

**DOI:** 10.3390/ani11040993

**Published:** 2021-04-01

**Authors:** Lucia Lazarowski, Bart Rogers, Sarah Krichbaum, Pamela Haney, Jordan G. Smith, Paul Waggoner

**Affiliations:** 1 Canine Performance Sciences, Auburn University College of Veterinary Medicine, Auburn, AL 36832, USA; ber0003@auburn.edu (B.R.); szk0138@auburn.edu (S.K.); wiltpam@auburn.edu (P.H.); waggolp@auburn.edu (P.W.); 2 Department of Psychological Sciences, Auburn University, Auburn, AL 36832, USA; jag0125@auburn.edu

**Keywords:** detection dog, behavior test, predictive validity, working dog

## Abstract

**Simple Summary:**

The ability to predict a puppy’s future working success is important for working dog programs to maximize the number of dogs placed in service and allocate resources to puppies most likely to succeed. This study evaluated the utility of a behavioral test for candidate detection dog puppies. Agreement between scores from different observers was high; demonstrating the reliability of the test. Scores based on dogs’ performance in the test were also consistent with scores derived from questionnaires completed by the dog’s trainers; demonstrating the validity of the test’s measures. Finally; puppies’ performance in the test predicted future selection as a working dog as early as three months of age. The results indicate that the behavioral test is useful for evaluating the potential success of detection dog puppies.

**Abstract:**

Behavioral characteristics are the most influential factor in the success of a working dog. The need for highly capable detection dogs continues to rise; but reliable methods for early selection are lacking. The current study aimed to assess the reliability and validity of a behavioral test for assessing detection dog suitability. A cohort of candidate detection dog puppies (*n* = 60) were tested at 3; 5; and 11 months of age; as well as at the completion of training at approximately one year. Tests were designed to assess important detection dog behavioral characteristics such as search ability and fearfulness. Inter-rater reliability was high between independent observers. Convergent validity was demonstrated by comparing Principal Component Analysis (PCA) scores from the behavior test to trainer ratings using the Canine Behavioral Assessment and Research Questionnaire (C-BARQ) and a survey of detection dog traits. Performance on the behavior test predicted adult selection as a detection dog as early as 3 months. The methods reported will be valuable for improving selection measures and enhancing collaborations across breeding programs in order to increase the availability of highly capable detection dogs.

## 1. Introduction

The most effective method currently available for locating explosives are trained detection dogs [1]. Widespread recognition of detection dogs’ efficacy, along with rising threats to global and homeland security, have led to an increase in their demand and a strain on the availability of dogs capable of performing such tasks [2]. The challenge in meeting this demand is further compounded by the rigorous behavioral standards required of an operational detection dog, resulting in high rates of attrition from training programs or early discharge from deployment [3,4,5].

Behavioral assessments have been developed and validated for guide and assistance dogs (e.g., [6,7,8]) and military/police dogs [9], but have been less explored for single-purpose detection dogs [10]. While there is some overlap in desirable behavioral characteristics across different types of working dogs, the constellation of critical behavioral traits of a successful single-purpose detection dog is likely unique [11]. For example, while lack of fearfulness and high trainability are important traits for most working dogs, detection dogs are also required to exhibit a strong intrinsic desire to hunt and work independently in a variety of dynamic environments [3].

The former United States’ Transportation and Security Administration (TSA) Canine Breeding and Development Center (CBDC) implemented a standardized behavior test for the program’s purpose-bred detection dogs, originally developed by the Australian Customs and Border Patrol detection dog breeding and development program [12]. The test was designed to evaluate domains considered most critical for a detection dog, sensitivity to novel stimuli and searching/retrieving abilities, at several time points across the first year of life. In an examination of the TSA CBDB test, McGarrity et al. (2016) found that improvements in behavior ratings across a puppy’s first year of life were predictive of selection into the training program, but scores given at a single time point were not [13]. Given the time, resources, and costs invested in rearing and preparing a dog for a future detection role, the economic and welfare implications of early release are significant [14]. Therefore, the ability to identify dogs with the behavioral repertoire necessary for detection tasks as early as possible is critical. However, few studies have found puppy tests to be predictive of adult behavior, with predictive ability increasing with age tested [15,16,17,18,19,20,21,22]. The lack of predictive power of tests administered at earlier time points may be due to the considerable hormonal and neurological changes that occur across early development [7]. On the other hand, early behaviors showing associations with adult behavior likely have a stronger genetic component and are more resilient to environmental influence. For example, some studies have shown that behavioral tests reflecting prey-drive and fearfulness, traits known to be heritable [23,24,25], were predictive of working dog outcomes as early as 7–12 weeks of age [17,19].

In addition to early identification of a dog’s future suitability as a detection dog, which can inform training decisions or career changes and increase program efficiency, standardized evaluation methods will be critical to increasing the availability of quality candidates through initiatives such as large-scale breeding cooperatives [2]. Consistent scoring schemes would allow for the sharing of data and breeding stock across programs, including the use of estimated breeding values for behavioral traits to enhance breeding decisions [2,26]. For example, the International Working Dog Registry (IWDR) is a recently developed database designed for working dog programs to store, track, and share phenotypic data, which will require data from a large number of dogs using the same scoring system [2].

The purpose of this study was to evaluate the validity and reliability of a behavioral test for selecting single-purpose detection dogs, and to determine its predictive validity from early ages. Proposed criteria for judging behavioral tests for working dogs include inter-rater reliability (IRR), test–retest reliability, and predictive validity [10,27]. Determining whether a test is reproducible (i.e., reliability) is one of the most critical aspects in validating a canine behavior test [28]. To this end, we evaluated IRR by comparing the scores from a live evaluator to those of additional independently observers. Intra-rater reliability (i.e., consistency across repeat observations within the same observer) was deemed unnecessary as inter-rater is suggested to be the stronger of the two types, and test–retest reliability was omitted due to potential changes in behavior due to repeated exposure to the test [28]. Construct validity is important for determining how well a test measures what it is purported to be measuring, which can be assessed by determining the strength of convergence with a separate well-established behavioral measure taken at the same time [28]. This type of convergent (also referred to as concurrent) validity is considered especially critical to the validation of behavioral assessments for young animals, because a failure to predict future behavior could be due to developmental changes rather than the test not being a valid measure of behavior [29]. We compared scores in the behavior test to scores derived from previously developed questionnaires of canine behavior that reflected traits considered important for detection dogs [30,31,32]. Finally, we assessed predictive validity by determining the association between dogs’ scores on the behavior test and their future outcome from the training program.

## 2. Materials and Methods

### 2.1. Subjects

Dogs belonged to the Auburn University College of Veterinary Medicine (AUCVM) Canine Performance Sciences (CPS) detection dog breeding program. The sample included in this study consisted of all dogs in the AUCPS program born between December 2018 and October 2019, resulting in 60 dogs (27 F/33 M) from eight different litters. Breeds included purebred Labrador retrievers (*n* = 48) and Labrador retriever X German wirehaired pointer crosses (F1b generation, *n*= 12). All activities were approved by the Institutional Animal Care and Use Committee of Auburn University (# 2019-3489 and # 2019-3490). Dogs in the CPS program are born and reared under identical conditions and experience standardized development, socialization, and training protocols. From birth through 7 weeks, puppies remain with the dam and litter in the CPS nursery and experience daily intensive socialization in the form of thorough handling and exposure to a variety of sights, sounds, and novel stimuli (e.g., [33]). At 7 weeks, puppies move to indoor/outdoor runs and begin formal training. From 6–10 months, puppies are placed in partnering prison programs for intermediate training (introduction to odor discrimination and searching), after which they return to CPS for approximately one month of advanced detection training (see [34] for detailed description on each phase). At the completion of training, dogs are presented to third-party customers who perform their own independent evaluation of the dog and make a determination of selection. Exceptions to this process are dogs selected by CPS to retain in the breeding colony, and dogs that are demonstrably incapable of performing detector dog tasks (i.e., showing extreme fear or apathy that would immediately disqualify them from the evaluation process). For the purpose of this study, dogs selected by a customer and breeders were categorized as ‘selected’. Dogs that were not presented to customers due to the unlikelihood of selection, or were presented and subsequently rejected for behavioral reasons, were categorized as ‘rejected’ (final numbers for each category reported below).

### 2.2. Procedure

#### 2.2.1. Behavioral Evaluation

All dogs underwent a standardized behavioral evaluation at 3 months (mean: 3.31 mo), 5 months (mean: 5.75), 11 months (mean: 11.55), and at the completion of final training (mean: 14.39 months). The evaluation was a revised version of a previous iteration [34], and consisted of tests similar to those traditionally used to evaluate detection dog suitability (e.g., [13,35,36]). The evaluation was composed of three separate parts, with several subtests within each part: (1) Performance Test, consisting of exercises designed to measure characteristics related to searching abilities and reward engagement, (2) Emotional Reactivity Test (ERT), in which dogs are confronted with a series of provocative stimuli, and (3) Environmental Test, a walkthrough in a natural dynamic environment resembling real-world scenarios. The evaluation was generally the same across ages with slight variations due to differences in test location and the dog’s age, increasing in difficulty at each time point. To minimize effects of repeated exposure, the tests were performed in different locations and with novel stimuli as much as feasible. The order of testing was the same for all dogs at each time point, but varied across time points. Testing began around 08:00 and was typically completed in the same day, weather permitting. Three individuals participated in all evaluations: the dog’s trainer who handled the dog throughout the test (handler), a senior CPS trainer who live-scored the dog throughout the test (evaluator), and a videographer who recorded all testing using a GoPro Hero5 camera. A second senior CPS trainer was present for a portion of the evaluations and independently live-scored the dogs. All individuals present remained behind the dog and handler throughout the tests.

##### Performance Test

The performance part of the test measured characteristics specific to training and ability in odor detection, and therefore involved exercises measuring dogs’ engagement with a toy reward and the handler as well as performance in a search task. Procedures for each subtest are described below, and scoring definitions are listed in Table S1.

- *Reward arousal*: the evaluator tossed a toy approximately 1 m away from the dog while the handler held the dog on leash for 10, 15, 20, or 25 s (increasing across time points). At the completion of the interval, the handler released the dog to retrieve the reward and then engaged the dog in brief tug-of-war upon return.
- *Reward focus* (performed at all ages except 3 months): the handler showed the dog the toy and held it up in front of the dog for approximately 3 s, at which point an object (5M: set of keys, 11M: 2-gallon plastic bucket, Final: metal dustpan) was dropped on a concrete surface behind the dog.
- *Reward persistence*: the evaluator showed the dog the toy and placed it inside a closed clear acrylic box (36 cm × 24 cm × 24 cm; H × L × W). The dog was observed for 10, 15, 20, or 25 s (increasing across ages).
- *Search test*: following the reward tests, an indoor search exercise was performed consisting of three hidden targets planted throughout the interior of a large warehouse-type building. For 3- and 5-mo old puppies, the target was a familiar toy (e.g., KONG^®^ Puppy, Wubba™, and Squeezz Jel, ChuckIt!^®^ ball and tugs). For the Final evaluation, the target was the odor which they had been trained to detect during final training (Double Base Smokeless Powder). The search portion of testing was not conducted at 11 months as dogs were transitioning from searching from toys to target odors at this point. The handler led the dog into the building and released the dog to search off-leash. The handler walked with the dog from one location to the next, providing minimal guidance and direction. When the dog located the target, the handler rewarded the dog with brief tug-of-war and then removed the reward to continue with the next search. If the dog became distracted, lost focus, or had difficulty locating the target, the handler provided prompts as needed (taken into consideration in the scoring). Hunt, Air Scenting, Task Engagement, and Working Arousal were scored across the three searches. Additionally, Handler Engagement and Possession were measured across all interactions with the handler or and/or reward.

##### Emotional Reactivity Test

The ERT evaluated detection dogs’ reactions to strange or startling stimuli and was similar to that used by Sherman et al. (2015) [36]. The version used here was designed to be shorter to allow for completion of the entire evaluation in the same day. The ERT was conducted in an enclosed indoor space where the dog navigated a circuit encountering a series of potentially fear-eliciting stimuli. Stimuli were chosen based on those commonly used for working dog assessments (e.g., [6,36,37,38,39]) and differed at each time point (see Table S2 for specific stimuli at each time point):

- *Novel Object*: A stationary animal-like statue
- *Sudden Appearance*: A person or object suddenly appearing in the dog’s path
- *Animated Object*: A remote-controlled object that becomes activated as the dog approaches it
- *Acoustic Startle*: A sudden and loud sound

No rewards were used during the ERT, and handler prompts were only given if the dog demonstrated difficulty continuing (taken into account in the scoring). Reactions to each stimulus were scored separately, using scoring definitions adapted from the Behavior Checklist (BCL), a validated behavior scoring system used to assess aspects of behavior important for working dogs, utilized by the IWDR [40] (Table S3).

##### Environmental Test

The Environmental Test was similar to that used by TSA’s former Canine Breeding and Development Center [13], and consisted of the handler walking the dog (on-leash) through a high-stimulus environment (e.g., different areas on the University’s campus and the city’s downtown district). The test was designed so that the dog encountered various challenges associated with operational environments such as unfamiliar objects (e.g., fire hydrants, trash cans), different types of surfaces (e.g., slick floors, grates) and stairs (e.g., solid, open-back, grated), pedestrian and vehicle traffic, and noises. No rewards were used during the walkthrough, and handler prompts were only given if the dog demonstrated difficulty continuing (taken into account in the scoring). Scores were given for the following items, using scoring definitions based on the BCL [40]: Anxious in Unfamiliar Situations, Fear of Traffic, Fear of Strangers, Noise Fear, Fear of Underfootings, Fear of Stairs, Fear on Elevation, Body Sensitivity to Object Contact (Table S4).

##### Tractability Rating

At the completion of the evaluation, the evaluator assigned the dog a ‘Tractability’ score reflecting the dog’s overall suitability for detection work, similar to the IWDR ‘Comparison Score’. Tractability was scored on a 1–5 scale: 1: dog is poorly suited for work and is recommended for adoption; 2: dog is difficult to train or manage, has limited placement options; 3: dog is trainable and manageable with some effort, capable of standard detection tasks; 4: dog is easily trainable and manageable, learns new tasks with few trials and little direction, easily comes under control of new situations and environments, is well-suited for any role; 5: dog is completely environmentally sound, consistent, manageable, highly responsive to training, and flexibly adaptable to new situations (e.g., breeder quality).

#### 2.2.2. Questionnaires

To assess convergent validity, we selected two questionnaires previously developed and utilized for evaluating dog behavior for comparison to scores from the behavioral evaluation. Questionnaires were selected for relevance to behaviors measured in the evaluation. For comparison to behavioral measures of trainability and temperament, we used The Canine Behavioral Assessment and Research Questionnaire (C-BARQ©, www.cbarq.org (accessed on 31 March 2021)) [30]. The C-BARQ is a validated and widely used behavioral assessment providing scores for subscales measuring trainability, social and non-social fearfulness, aggression, and other miscellaneous behaviors. For the purpose of our study, we selected C-BARQ subscales that reflected behaviors measured in the evaluation. Further, several C-BARQ subscales require observing dogs in situations not applicable to our population (i.e., in a home environment). For comparison to the Performance Test, we used the C-BARQ subscale Trainability (8 items); however, “obeys the sit command immediately” and “obeys the stay command immediately” were not applicable for the youngest groups (< 11 months) as these commands are not taught at these ages. For comparison to the ERT, we used the C-BARQ subscale Non-Social Fear (6 items). For comparison to the Environmental Test, we used the C-BARQ subscales Non-Social Fear, Stranger-Directed Fear (4 items; however, the item “when an unfamiliar person visits your home” was not applicable and was not able to be scored), and miscellaneous item Fear of Stairs rating. For validation of the Tractability Rating, we used the C-BARQ subscales of Trainability, Non-Social Fear, and Stranger-Directed Fear.

Questions were answered using either a frequency scale (0–4 scale: Never, Seldom, Sometimes, Usually, Always) or a severity scale (0–4 scale: Absent to Severe presence of a behavior), with an option for “not observed/not applicable”. For each subscale, scores for all items pertaining to the subscale were averaged.

The C-BARQ has been validated in pet and some working dog populations including assistance and guide dogs [6,26,30], but may not capture important traits unique to a detection dog such as searching ability. Therefore, we also asked trainers to rate the dogs on a set of traits previously found to be important for scent detection dogs [32,41,42]: Obedience to human command, Boldness, Playfulness, Tendency to hunt by smell alone, Stamina, Ability to learn from being rewarded, Interest in toys or objects, Acuity of sense of smell, and Motivation to retain possession of an object. Each characteristic was rated on a 1–5 scale from extremely low to extremely high, and a mean ‘Detection Dog Attributes’ score was calculated for each dog.

Questionnaires were completed by each dog’s trainer within one week prior to the evaluation, to ensure responses were not influenced by the dog’s performance in the evaluation [32]. Questionnaires were completed at each time point, except for 11 months due to the behavioral evaluation being performed on the day following arrival back to CPS.

### 2.3. Statistical Analyses

#### 2.3.1. Inter-Rater Reliability

We evaluated IRR in two ways. First, we compared scores between the two senior CPS trainers for all evaluations in which both were present. Both trainers had over 12 years of experience working with detection dogs, and were familiar with the evaluation procedure. The primary evaluator was familiar with the dogs but was not their main trainer, and the second evaluator was not familiar with the dogs in the current sample. Second, an independent evaluator not present during the evaluations scored a random selection of 20% of the evaluations at each time-point from video. This evaluator was a behavior researcher experienced in evaluating working dog behavior, but was not familiar with the dogs or the evaluation. Kendall’s coefficient was calculated for all IRR analyses. All subsequent analyses used the primary evaluator’s scores.

#### 2.3.2. Principal Component Analyses

A principal component analysis (PCA) was performed for each test to reduce the number of variables and identify meaningful underlying dimensions of behavior to be used in subsequent analyses [13]. To be able to evaluate early predictive validity, separate PCAs were performed for each time point. Sampling adequacy of PCA was confirmed by Bartlett’s sphericity tests and Kaiser–Meyer–Olkin (KMO) measures, and the correlation matrix of test scores was deemed appropriate for PCA [43]. Determination of final components was based on eigenvalues >1, with Varimax rotation, and loading of 0.50 or higher considered significant given the sample size [7,43,44]. PCA-derived component scores, variables loading strongly onto more than one component, and uncorrelated variables were used in subsequent analyses.

#### 2.3.3. Convergent Validity

Convergent validity was assessed by a correlation (Pearson for normally distributed data and Spearman’s rank for non-normal data, assessed by Kolmogorov–Smirnov tests) between the PCA-derived component scores and corresponding questionnaire scores from the same time point. Scores from the Performance Test were correlated to C-BARQ Trainability and the Detection Dog Attributes score. Scores from the ERT were correlated to C-BARQ scores for Non-social fear. Scores from the Environmental Test were correlated to C-BARQ scores for Stranger-directed fear, Non-social fear, and Fear of Stairs. Finally, the Tractability rating was correlated with C-BARQ Stranger-Directed Fear, Non-Social Fear, Trainability, and the Detection Dog Attributes score.

#### 2.3.4. Predictive Validity

To determine predictive validity of the behavioral evaluations, separation binary logistic regressions were performed for each test at each time-point. PCA-derived scores and uncorrelated variables were entered as fixed factors with final outcome from the training program (selected/rejected) as the dependent variable. To determine potential effects of sex, we first ran a Generalized Linear Model (GLM) with sex as a fixed factor and PCA component scores/uncorrelated variables as dependent variables. Where a significant effect of sex on a variable was found, the interaction between sex and the variable was included in the logistic regression to determine whether predictive validity of a measure differed between the sexes. 

## 3. Results

### 3.1. Inter-Rater Reliability

Table 1 shows the IRR for each of the tests at each time point. A second evaluator was present for 24 of the 3-months (*n* = 513 individual observations) and 25 of the 5-months (*n* = 672 individual observations) evaluations. Additionally, an independent evaluator scored a random selection of 20% of the evaluations for each of the time points from video, resulting in 277, 311, 213, and 267 observations across the four time points, respectively. Agreement between the two live scorers was very good and statistically significant across all of the measures, and was high and statistically significant for all measures except tractability between the live scorer and the video scorer (Table 1).

### 3.2. Principal Component Analyses

#### 3.2.1. Performance Test

Table S5 shows PCA loadings for the Performance Test at each time point. At 3 months, Handler Engagement, Reward Arousal, and Working Arousal did not have any correlations >0.5 and were removed and retained as individual items. The remaining five variables loaded strongly onto a single component in the PCA, explaining 68.90% of the total variance (Bartlett’s sphericity *x*^2^ = 188.792, *p* < 0.001, KMO = 0.785). This component had high loadings of variables related to searching and interaction with the reward and was labeled ‘3M Work Drive’. At 5 months, Reward Arousal and Working Arousal did not have correlations >0.5 and were removed and retained as individual items. The remaining seven variables showed significant correlations and loaded strongly onto two components (*x*^2^ = 195.285, *p* < 0.001, KMO = 0.815). The first component explained 50.293% of the total variance and reflected high scores related to searching and persistence and low scores for handler engagement, and was labeled ‘5M Work Drive’. The second component explained 17.62% of the total variance and reflected high scores for handler and reward engagement and was labeled ‘*5M Reward Engagement*’. At 11 months*,* Handler Engagement and Reward Arousal did not have any correlations > 0.5 and were removed and retained as individual items. The remaining three variables showed significant loadings onto a single factor explaining 59% of the total variance (*x*^2^ = 24.406, *p* < 0.001, KMO = 0.585), reflecting high scores for reward persistence and engagement and was labeled ‘11M Reward Engagement’. In the Final Performance Test, Handler Engagement, Reward Arousal, and Working Arousal did not have any correlations >0.5 and were removed and retained as individual items. The remaining five variables loaded strongly onto a single component explaining 63.41% of the total variance (*x*^2^ = 126.143, *p <* 0.001, KMO = 0.811). This component reflected high scores for searching and reward interaction and was labeled ‘Final Work Drive’.

#### 3.2.2. Emotional Reactivity Test

Table S6 shows PCA loadings for the ERT at each time point. At 3 months, 11 mo, and Final, all four ERT items loaded onto a single component explaining 52.75% (*x*^2^ = 188.792, *p <* 0.001, KMO = 0.675), 52.87% (*x*^2^ = 39.634, *p <* 0.001, KMO = 0.682), and 61.63% (*x*^2^ = 60.669, *p <* 0.001, KMO = 0.767) of the total variance, respectively. At 5 months, Novel Object did not correlate significantly with the other items and was removed and retained as an individual item. The three remaining subtests loaded onto a single component explaining 64.97% of the total variance (*x*^2^ = 36.56, *p <* 0.001, KMO = 0.645). For all ages, the component reflected minor reactions and quick recovery to the stimuli and was labeled ‘Emotional Reactivity’.

#### 3.2.3. Environmental Test

Table S7 shows PCA loadings for the Environmental Test at each time point. At 3 months, Fear of Stairs loaded strongly onto more than one component, and Fear of Underfootings, Fear of Strangers, and Fear on Elevation did not have any correlations >0.5, and therefore were removed and retained as individual items. The PCA on the remaining items suggested a single-component solution explaining 67.53% of the total variance (*x*^2^ = 92.291, *p <* 0.001, KMO = 0.799). The component reflected sensitivity to sounds and unfamiliar situations and was labeled ‘3M Environmental Sensitivity’. At 5 months, Fear of Strangers and Fear of Underfootings did not have any correlations >0.5 and were removed and retained as individual items. The remaining six variables loaded strongly onto two components explaining 67% of the total variance (*x*^2^ = 103.689, *p <* 0.001, KMO = 0.675). Component 1 explained 40.81% of the total variance and reflected sensitivity to unfamiliar sounds and situations and was labeled ‘5M Environmental Sensitivity’. Component 2 explained 27.10% of the total variance and reflected hesitation on elevation, stairs, and in narrow spaces and was labeled ‘5M Proprioception’. At 11 mo, Fear of Underfootings did not have any correlations >0.5, and Body Sensitivity to Object Contact loaded strongly onto more than one component, therefore both were removed. The remaining six variables loaded strongly onto two components explaining 65% of the total variance (*x*^2^ = 55.317, *p <* 0.001, KMO = 0.594). Component 1 explained 40% of the total variance and reflected sensitivity to noises, traffic, elevated surfaces, and stairs, and was labeled ‘11M Environmental Confidence*’*. Component 2 explained 25% of the total variance and reflected hesitation around unfamiliar people and places and was labeled ‘11M Boldness’.

Due to restrictions to areas normally used for the Environmental Test during the COVID-19 pandemic, four litters did not receive the full evaluation at the Final time point. Therefore, because PCA excludes data with any missing cases, the PCA at this time point was based on data from 26 dogs. However, the KMO was too low (0.550) for PCA, therefore we used the average score of all non-social items and the raw score for Fear of Strangers and Fear of Stairs.

### 3.3. Convergent Validity

Performance Test PCA components were compared to C-BARQ Trainability and the Detection Dog Attributes score at each time point, with the exception of 11M for which questionnaires were not collected. 3M and Final Work Drive showed moderate to strong correlations with both C-BARQ Trainability (3M: *r*(58) = 0.399, *p* = 0.002; Final: *r*(51) = 0.515, *p* < 0.001) and Detection Dog Attributes (3M: *r*(58) = 0.508, *p* < 0.001; Final: *r*(52) = 0.707, *p* < 0.001). 5M Work Drive strongly correlated with Detection Dog Attributes (*r*(53) = 0.537, *p* < 0.001) but not C-BARQ Trainability (*r*(53) = 0.021, *p* = 0.811), and 5M Reward Engagement did not correlate with either (*p*s > 0.69).

The ERT component score for each age was compared to the corresponding C-BARQ Non-Social Fear subscale score. Correlations were significant with moderate strength for all time points (3M: *r*(58) = 0.435, *p* = 0.001; 5M: *r*(53) = 0.455, *p* < 0.001 Final: *r*(51) = 0.406, *p* = 0.003).

Environmental Test scores were compared to C-BARQ subscale scores for Stranger-Directed Fear, Non-Social Fear, and Fear of Stairs. The 3M Environmental Test component showed moderate to strong correlations with all three C-BARQ subscales (Stranger-Directed Fear: *r*(58) = 0.496, p < 0.001; Non-Social Fear: *r*(58) = 0.549, *p* < 0.001); Fear of Stairs: *r*(51) = 0.473, *p* < 0.001). At 5M, the first component (Environmental Sensitivity) did not correlate with any C-BARQ scores, but the second component (Proprioception) showed moderate and weak correlations with C-BARQ scores for Non-Social Fear (*r*(53) = 0.415, *p* = 0.002) and Fear of Stairs (*r*(53) = 0.286, *p* = 0.034), respectively. In the Final test, there was a moderate correlation between the Fear of Strangers evaluation score and C-BARQ Stranger-Directed Fear, *r*(37) = 0.449, *p* < 0.006, as well as between the Fear of Stairs evaluation score and the C-BARQ Fear of Stairs rating, *r*(53) = 0.321, *p* = 0.019; the score for non-social items did not correlate with any of the C-BARQ scores (*p*s > 0.11).

The Tractability rating was compared to C-BARQ Trainability, Stranger-Directed Fear, and Non-Social Fear subscales, as well as the Detection Dog Attributes score. At 3M, Tractability correlated weakly with Detection Dog Attributes (*r*(58) = 0.294, *p* = 0.022), and nothing else (*p*s > 0.124). 5M Tractability correlated with Detection Dog Attributes (*r*(53) = 0.405), *p* = 0.002), C-BARQ Social-Directed Fear (*r*(53) = 0.278, *p* = 0.040), and C-BARQ Non-Social Fear (*r*(53) = 0.381, *p* = 0.004), but not C-BARQ Trainability (*p* = 0.647). Final Tractability correlated with all questionnaire scores (C-BARQ Social-Directed Fear: *r*(53) = 0.437), *p* < 0.001; Non-Social Fear: *r*(53) = 0.475, *p* < 0.001, Trainability: *r*(53) = 0.315, *p* = 0.022; Detection Dog Attributes: *r*(53) = 0.369, *p* = 0.007).

### 3.4. Predictive Validity

Of the initial sample of 60 prospective detection dogs, 28 (9 F/19 M) were categorized as selected (23 selected as an operational detection dog and five selected as a breeder). Of the dogs categorized as ‘rejected’, six were adopted as pets and the remaining 26 were employed non-operationally by AUCPS (i.e., utilized for odor detection tasks in a non-operational laboratory setting). Predictive validity results for each of the tests are summarized in Table 2.

#### 3.4.1. Performance Test

**Figure 1:**
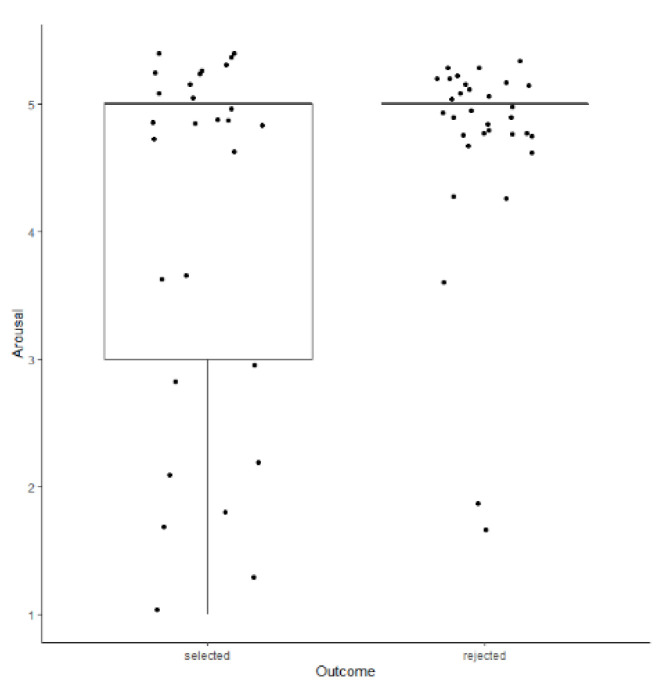
Distribution of ratings for Working Arousal (1–5 scale) in the Performance Test for 3-months-old puppies as a function of eventual program outcome. Horizontal lines represent medians, boxes represent the interquartile range, and whiskers represent the range of values within 1.5 × interquartile range. Dots are individual data points, jittered to reduce over-plotting.

There were no effects of sex on any of the 3M Performance Test variables (*p*s > 0.34). Working Arousal was predictive of outcome, where lower scores (higher arousal) was predictive of selection for a working role as an adult (*z* = −1.975, *p* = 0.048; Figure 1). 3M Work Drive and remaining uncorrelated variables (Handler Engagement, Reward Arousal) were not predictive of outcome (*p*s > 0.37).

At 5 months, males scored worse (i.e., higher arousal) than females on Reward Arousal (*t* = −2.02, *p* = 0.048), but there was no interaction between sex and Reward Arousal on outcome. There were no sex effects on any of the other Performance Test variables (*p*s > 0.192), and none of the 5M Performance Test variables were predictive of outcome (*p*s > 0.17).

At 11 months, males scored significantly higher than females on 11M *Reward Value* (*t* = 2.358, *p* = 0.022), and significantly lower on Handler Engagement (*t* = −2.872, *p* = 0.006), however none of the interactions were significant (*p*s > 0.131). None of the 11M Performance Test measures were predictive of outcome (*p*s > 0.125).

At the Final time point, males scored higher than females on Work Drive and Reward Persistence (*p* = 0.022 and 0.026, respectively). Interactions between the variables with sex on outcome were not significant. Higher scores for Reward Persistence (*z* = 2.765, *p* = 0.005) and lower scores for Handler Engagement (z = −2.34, *p* = 0.019) were predictive of future selection. Work Drive, Reward Arousal, and Working Arousal were not predictive (*p*s > 0.07).

#### 3.4.2. Emotional Reactivity Test

**Figure 2:**
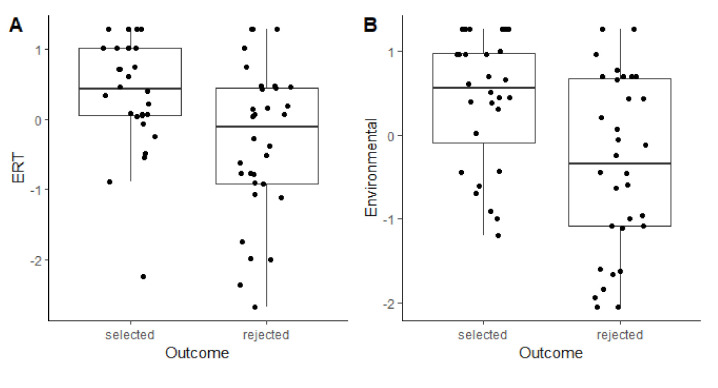
Distribution of component scores from the Emotional Reactivity Test (**A**) and Environmental Test (**B**) for 3-months-old puppies as a function of eventual program outcome. Horizontal lines represent medians, boxes represent the interquartile range, and whiskers represent the range of values within 1.5 × interquartile range. Dots are individual data points, jittered to reduce over-plotting.

At 3 months, there was no effect of sex on Emotional Reactivity (*p* = 0.58). Higher scores (i.e., milder reactions and quicker recovery) were significantly predictive of future selection (*z* = 2.519, *p* = 0.011; Figure 2A).

At 5 months, there was no effect of sex on Emotional Reactivity (*p* = 0.804) or uncorrelated variables (Novel Object, *p* = 0.265). 5M Emotional Reactivity was not predictive of final outcome (*p* = 0.06), but higher scores on the Novel Object subtest was predictive of future selection (*z* = 2.093, *p* = 0.036).

At 11 months, males scored higher than females on Emotional Reactivity (*t* = 2.08, *p* = 0.042), with no interaction between sex and Emotional Reactivity on outcome (*p* = 0.692). Higher scores on the 11M Emotional Reactivity were predictive of future selection (*z* = 3.345, *p* = 0.001).

There was no effect of sex on Final Emotional Reactivity (*p* = 0.076). Higher scores were predictive of future selection (*z* = 3.32, *p* = 0.001).

#### 3.4.3. Environmental Test

At 3 months, males scored significantly better than females on Fear of Stairs (*t* = 2.719, *p* = 0.008), but the interaction with outcome was not significant. There was no effect of sex on 3M Environmental Sensitivity or the other uncorrelated variables (*p*s > 0.168). Puppies scoring higher on 3M Environmental Sensitivity were more likely to be selected as a working dog in the future (*z* = 2.67, *p* = 0.007).

At 5 months, males scored significantly better than females on 5M Environmental Sensitivity (*p* = 0.006), and the interaction with sex on outcome was not significant. Higher scores on 5M Environmental Sensitivity were predictive of future selection (*z* = 2.655, *p* = 0.007), while the remaining variables were not (*p*s > 0.16).

At 11 months, males scored significantly better than females on Fear of Underfootings, (*t* = 3.732, *p* = 0.001), with no interaction with sex on outcome (*p* = 0.765) and no sex effects on remaining variable (*p*s > 0.065). Higher scores on both components from the 11M Environmental Test significantly predicted future selection (*z* = 2.344, *p* = 0.019; *z* = 2.578, *p* = 0.009), and uncorrelated variables were not predictive (*p* = 0.07).

At the Final time point, males scored higher than females on the non-social average (*t* = 2.584, *p* = 0.0126), and Fear of Stairs (*t* = 2.876, *p* = 0.005), with no significant interactions. The average score of the non-social items was predictive of outcome (*t* = 2.56, *p* = 0.01), while Fear of Strangers and Fear of Stairs were not (*p*s > 0.07).

#### 3.4.4. Tractability Rating

Neither 3M, 5M, or Final Tractability were affected by sex (*p* = 0.53, 0.09, 0.249, respectively). At 11M, males scored higher than females (*t* = 0.2476, *p* = 0.001), however the interaction between sex and Tractability on outcome was not significant. 3M Tractability was not predictive of final outcome (*p* = 0.718), but all other time points were (5M: *z* = 2.19, *p* = 0.028; 11M: *z* = 3.721, *p* = 0.0002; Final: *z* = 3.626, *p* = 0.0003).

## 4. Discussion

Behavioral deficiencies are the most common cause for early release from training or active deployment across different working dog programs. Given the increasing demand for highly capable detection dogs and the time and costs involved in their preparation, the ability to predict their success as early as possible is critical. The current study sought to validate a behavioral test for predicting future success as an operational single-purpose detection dog. A cohort of candidate detection dog puppies performed standardized evaluations at 3, 5, and 11 months of age, as well as a final evaluation at the completion of all training around 12–14 months of age. We found high consistency in scoring between different evaluators, convergence with other previously established assessments of dog behavior, and prediction of future outcome as a detection dog from as early as 3 months of age.

One of the most essential aspects of a test’s validity is its reproducibility, such as consistency in scores from one administration of the test to another, or between different scorers [28]. In this case, potential habituation or learning from repeated exposure to the test stimuli limits the ability to assess test-re-test reliability. We therefore evaluated reliability by comparing the scores between different evaluators. All evaluations were live-scored by the same experienced senior trainer, and when available, by a second experienced senior trainer. Agreement between the two trainers was high for all of the tests (0.80–0.90), exceeding the recommended acceptable minimum of 0.70 [28]. Additionally, we assessed IRR between the primary trainer’s live scores and an independent observer who scored 20% of the videos at each time point. IRR between these scorers was high and met the recommended criteria for the majority of the categories. The three instances for which the IRR was low was for Tractability at all time points except the final, suggesting that this measure may require more experience evaluating young dogs from this population, or that the definition lacks objectivity needed to score accurately. Indeed, Tractability is the only measure in the evaluation based on an overall subjective assessment of the dog’s suitability rather than based on a specific behavioral test, and furthermore, was not predictive of success at younger ages. Overall, results from the IRR analyses suggest that the procedures and definitions allow for objective and reliable scoring across different individuals with at least some experience in assessing canine behavior.

We next assessed convergence between the behavioral evaluation and previously established measures of canine behavior by comparing test scores to scores derived from questionnaires completed by the dogs’ trainers prior to the test. We selected the C-BARQ as it has been previously validated as a measure of canine behavior and frequently used in similar studies of working dog behavior [6,44,45,46], as well as a questionnaire rating traits specific to detection dogs. At each time point, correlations were significant for at least one of the two comparison questionnaires for all tests, though correlation coefficients were not as high as the recommended strength of 0.70–0.80 and not all items correlated. However, low levels of convergence between behavioral tests and questionnaires have been argued as evidence of validity due to the vast differences between the methods [29]. Additionally, the questionnaires used were either not designed for this population, or were designed for adult dogs. Indeed, the strength of the correlations increased across ages for all tests. It should also be noted that some items in the C-BARQ subscales used were not able to be scored, which could compromise the validity of the subscale [45].

Taken together, the results of the convergent validity analyses indicate that the current evaluation procedures are valid measures of canine behavior and detection ability. These findings suggest that the evaluations performed at a single time point and scored by a lesser or unfamiliar individual are reflective of scores based on trainers’ cumulative impressions of dogs’ behavior observed over an extended period of time working closely with the dog [28]. While questionnaire assessments can be a cost-effective and efficient way of evaluating canine behavior, accurate scoring depends on familiarity with the dog in a variety of situations. Thus, questionnaires may not be feasible during procurements performed by unfamiliar individuals, and if reported by the dog’s owner/trainer, requires the reporting to be honest and accurate [3,10].

Finally, we assessed whether the behavioral evaluations were predictive of future selection as a detection dog. We found that at least some measures from each subtest were predictive of future outcome from at least one time point Most importantly, the evaluation demonstrated predictive validity of future outcome as an adult as early as 3 months of age. Scores from the Performance Test were the least predictive across time points, with component scores from the PCAs (reflecting motivation to work and engage with a reward) failing to predict outcome at any time point. However, one of the uncorrelated items in the 3M test (Working Arousal) and two uncorrelated items from the Final test (Handler Engagement and Reward Persistence) were predictive of outcome. At face value, these findings could suggest that traits related to detection performance (as measured by this specific test) are not as important for detection dog success. This explanation is unlikely given previous findings that similar characteristics are related to success in different types of detection dogs [12,34,41,47]. It is also possible that the specific tests and definitions used to assess performance require further refinement and were not accurately capturing the desired traits, though convergence with the questionnaires suggests otherwise. Alternatively, there may not have been enough variability in our sample in relation to the performance domain. Furthermore, we previously reported that insufficient environmental soundness was the greatest reason for failure in this population, and that detection-related performance characteristics were predictive of type of detection career (e.g., person-borne versus standard explosives detection) but not outright selection [34]. Indeed, the fact that the vast majority of rejected dogs were suitable for in-house non-operational detection tasks illustrates that performance characteristics were not the cause for rejection. It is likely that detection-related traits have been more amenable to selective breeding, resulting in a homogenous sample in regard to these traits [34]. Further validation with more diverse external samples will be needed to confirm the relative significance of these traits for detection dog success.

Interestingly, some of the measures from the Performance Test that did predict final outcome were in the opposite of the presumed desirable direction. In the final evaluation, higher levels of Handler Engagement were associated with lower likelihood of success. While independence is generally considered to be important to a detection dog’s ability to work autonomously without influence from or dependence on the handler [34,48], this measure was added to the evaluation to capture dogs’ cooperativeness. For example, dogs need to be responsive to handler commands, and a willingness to cooperate is considered to reflect trainability [3]. We recently reported that dogs that make eye contact with the handler during an unsolvable task, interpreted as a communicative signal, are more likely to be selected for a future detection role [49]. On the other hand, dogs that followed a misleading human cue over conflicting available sensory information regarding the location of a hidden reward were less likely to be selected [50]. Taken together, desirable levels of sociability likely follow a bell-shaped trajectory, where optimal levels reflect a combination of independence and willingness to cooperate [3]. Therefore, the 1–5 scale for scoring this measure is likely inadequate to capture the optimal, likely mid-range, level of handler engagement. Alternatively, or additionally, high levels of handler engagement could reflect underlying anxiety and lack of confidence resulting in attachment behavior. Given that anxiety-related behavior was the primary reason for failure in this population, this interpretation is reasonable.

A similar inverse association was found between Working Arousal and future outcome in the 3M test, in that poorer arousal scores (i.e., higher arousal) were predictive of future selection. While arousal was generally conceived to be an undesirable behavior as it can interfere with problem-solving [51] and handling or management of the dog [3], it likely reflects underlying desirable characteristics such as higher motivation and desire to work. Further, high levels of arousal are common in working dog populations and it is not likely to be seen as an impasse by those selecting dogs [52]. Interestingly, this association was only seen at the 3-months time point, suggesting that differences in arousal during puppyhood may reflect important individual differences in motivation that are not as apparent with maturation. We previously reported a similar effect where puppies that exhibited more difficulty in an inhibitory control task, which we interpreted to result from increased arousal due to the difficult of the task, were more likely to be selected as a detection dog in the future [53]. Further work is needed to determine optimal levels of arousal for detection dogs that allow for sufficient energy and motivation to work, without compromising efficiency, management, and welfare. As with handler engagement, arousal is not likely to be accurately scored on a linear scale.

Environmental soundness was predictive of future outcome by both tests at all time points, corroborating previous findings of the importance of this domain in working dog success [9,54,55,56]. The validity of these measures was likely enhanced by the adoption of the previously validated IWDR Behavior Checklist scoring system. Our results indicate that the utility of the Behavior Checklist, initially designed for assistance and guide dogs, is generalizable to detection dog populations. While both the ERT and Environmental tests were predictive of final outcome at all ages, the ERT is a more rapid and standardized approach compared to the lengthier and more variable walkthrough which is subject to inconsistencies in environmental conditions (e.g., weather, traffic, people). As dogs experienced the battery of stimuli in the ERT in sequence, we are unable to determine whether the predictive value of the test is due to a global accumulation effect or to isolated effects of particular stimuli [8]. Further research could explore whether singular components of the ERT are more predictive than others such that unnecessary components can be removed, condensing the test to the most important features. Notably, in the 5M ERT, reactions to the novel object were not associated with the other parts of the test, and only this part of the test was predictive of final outcome. Further research could also explore whether particular features of the stimuli used are important, such as the use of animal-like objects with eyes.

Sex effects were observed for several of the measures where males scored higher than females on measures of work and reward motivation as well as environmental soundness. No sex effects were found for Tractability, and no sex effects emerged until the 5M time point. Further, sex effects were more prominent at the 11M time point where four measures differed between the sexes compared to the 5M and Final where only two measures differed. These age-related sex effects suggest that the differences may have been driven by periods of sexual development. A recent study found that behavioral disruptions coincided with phases of adolescence in female guide dogs, and suggests adolescence as another sensitive period during development [57]. It is therefore possible that female dogs in the current study were more affected than males during this period of hormonal change. We also found that males were more likely to be selected than females, in line with our previous findings [34] as well as a recent analysis of cadaver detection dog selection [58]. However, others have reported no sex-specific selection bias for detection dogs [13]. Regardless of the sex effects observed in the current study, there were no interactions between any of the measures and sex, indicating that sex differences did not impact the predictive validity of the evaluation measures. Nevertheless, effects of sex on trainability can vary by breed, warranting caution in generalizing findings to other breeds [59].

A limitation of the current study is the homogeneity of the sample concerning breed and pedigree. As discussed, it is possible that this population is unique with respect to certain performance-related traits due to selection from working lines as well as further selective breeding. Indeed, assessment of retrieving was removed from a previous iteration of the evaluation due to its inability to predict future outcome, likely due to a lack of variability within the population [34]. However, propensity to retrieve is widely considered an important trait for many working dogs and has been shown to be an early predictor of success [17]. Therefore, the importance of different traits is likely relative to the breed and population.

Another limitation is the use of outcome for the predictive models. While most studies of behavioral tests for working dogs use outcome as the definitive measure for validation, this measure is highly subjective and can fluctuate based on supply and demand [18]. Further, selection only captures success at that particular point in time, and behavioral problems leading to early release may develop later on [4]. Future research should strive to track dogs’ long-term success in the field which may be more informative than outcome measures reflecting the time of selection.

Finally, limitations regarding the practical validity of the results should be taken into consideration. While our results illustrated patterns of behavior that statistically predicted future outcome, the use of the test to make a definitive decision at a single time point about excluding a dog from further training is not likely. Therefore, the cost–benefit balance of performing the test should be considered. However, results of the test are likely to be valuable in other aspects, such as tailoring training to address performance deficiencies identified by the test as well as collecting and sharing phenotypic data.

## 5. Conclusions

Behavioral suitability is widely recognized as the most influential factor in the success of a detection dog [27,60], a lack of which is commonly reported as reasons for rejection or early release from training or deployment [5,34,54,56,61]. As the demand for capable detection dogs continues to rise, improved methods for selecting dogs is critical. This study reports on the validation of a behavioral test for predicting future suitability as a detection dog. Analyses demonstrated high agreement between independent observers (inter-rater reliability), consistency between test scores and trainer reports of the dogs’ behavior (convergent validity), and predictive validity of selection as an operational detection dog as early as three months. The methods reported will be valuable for improving selection measures and enhancing collaborations across breeding programs in order to increase the supply of highly capable detection dogs.

## Figures and Tables

**Table 1:** Kendall’s coefficients for inter-rater analyses between the primary live scorer and the two secondary scorers. Dashed lines indicate time points in which the second live scorer was not present.

	Kendall’s *W* Coefficient of Concordance							
	Performance Test		Emotional Reactivity Test		Environmental Test		Tractability Rating	
Time Point	between Two Live Scorers	between Live Scorer and Video	between Two Live Scorers	between Live Scorer and Video	between Two Live Scorers	between Live Scorer and Video	between Two Live Scorers	between Live Scorer and Video
3 months	0.924 ***	0.830 **	0.945 ***	0.914 ***	0.944 ***	0.818 ***	0.941 *	ns (*p* = 0.076)
5 months	0.899 ***	0.696 **	0.930 ***	0.957 ***	0.927 ***	0.825 ***	0.917 *	ns (*p* = 0.101)
11 months	-	0.835 **	-	0.877 **	-	0.892 ***	-	ns (*p* = 0.078)
Final	-	0.804 ***	-	0.900 **	-	0.827 ***	-	0.969 *

* *p <* 0.05, ** *p <* 0.01, *** *p <* 0.001, ns = not significant.

**Table 2:** Summary of results from the binary logistic regressions determining predictive validity of final outcome (selected/rejected) at each time point.

Test		Predictive?		
	3M	5M	11M	Final
Performance Test	✔	x	x	✔
Emotional Reactivity Test	✔	✔	✔	✔
Environmental Test	✔	✔	✔	✔
Tractability Rating	x	x	✔	✔

✔ indicates that at least one factor (either PCA component or individual item) from the test was a significant predictor of future selection as a detection dog (*p* < 0.05).

## Data Availability

The data presented in this study are available on request from the corresponding author.

## Supplementary Materials

The following are available online at https://www.mdpi.com/article/10.3390/ani11040993/s1, Table S1: Definitions and scoring for the Performance Test; Table S2: stimuli used in the ERT; Table S3: Definitions and scoring for the ERT; Table S4: Definitions and scoring for the Environmental Test; Table S5: PCA loadings for the Performance test variables at each time point; Table S6: PCA loadings for the ERT variables at each time point; Table S7: PCA loadings for the Environmental test variables at each time point.
